# Scope of Nursing Practice in Primary Health Care: defining a way forward *

**DOI:** 10.1590/1518-8345.0000.4840

**Published:** 2025-11-17

**Authors:** Bruna Moreno-Dias, Edwin Vicente C. Bolastig, Eduardo Benjamín Puertas-Donoso

**Affiliations:** 1 Pan American Health Organization, Department of Health Systems and Services, Human Resources for Health Unit, Washington, D.C., United States of America.; 2 Pan American Health Organization, Subregional Program Coordination, Caribbean, Bridgetown, Barbados.

**Figure:**
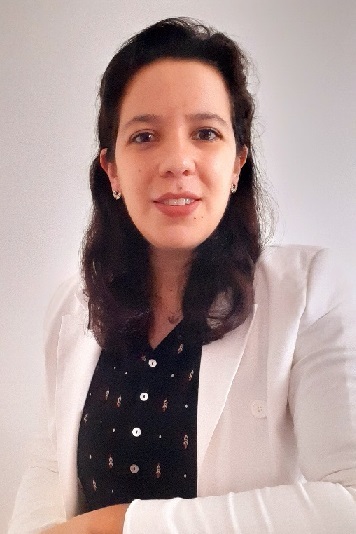

**Figure:**
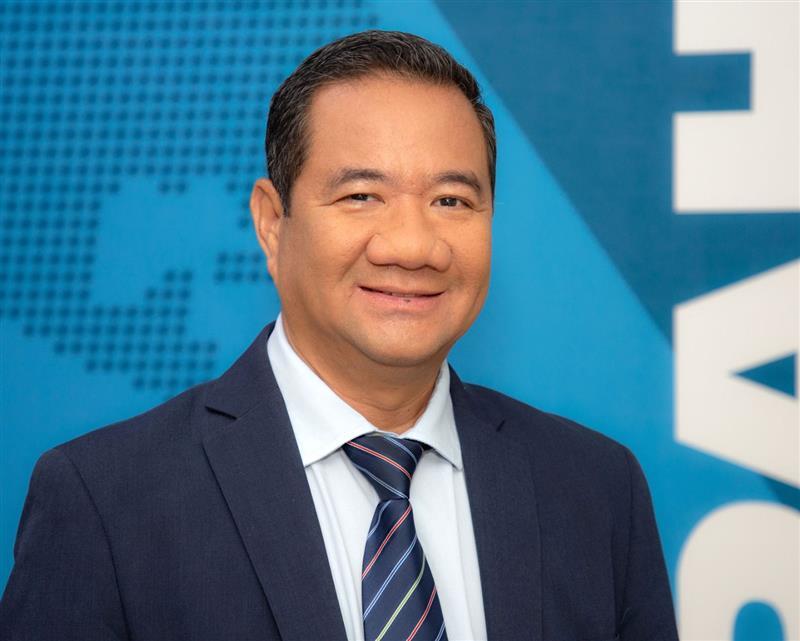

**Figure:**
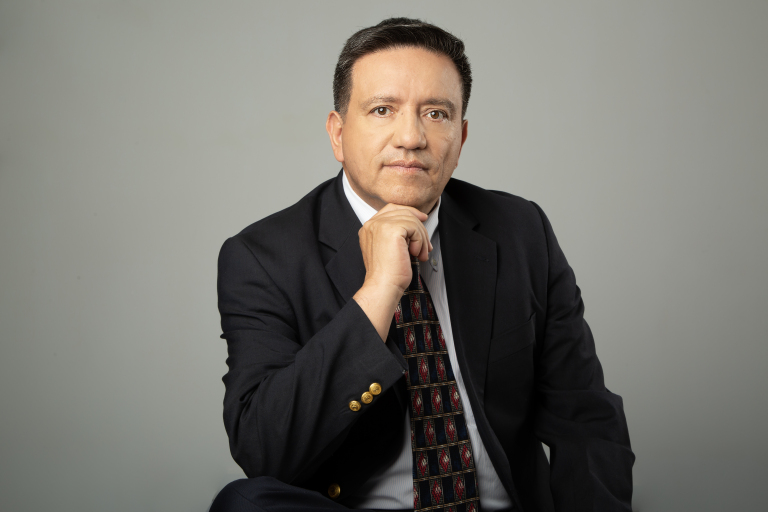

Primary Health Care (PHC) is recognized as the structural foundation of health systems, a comprehensive strategy for the organization and functioning of the health system, with the main objective of achieving universal health. Health systems based on PHC must offer a wide range of comprehensive services, which must be accessible, equitable, of high quality and comprehensive to meet the health needs of all people throughout their lives, provided by a workforce guided by collaborative practice in interprofessional teams^(1-2)^.

In this context, the health workforce is a fundamental component for the effectiveness, equity and resilience of health systems. It is also worth highlighting the essential role played by nursing personnel, due to their ubiquitous presence in the territories, their relationships with service users and the breadth of their clinical work^(2-3)^.

The Region of the Americas has approximately 7.4 million nursing personnel; however, it faces persistent challenges related to the equitable distribution of these professionals, the training and qualification of this workforce, the updating of regulatory frameworks and the strengthening of the scope of practice in complex and challenging contexts^(3)^.

In response to this scenario, Pan American Health Organization (PAHO) “Policy on the Health Workforce 2030: Strengthening Human Resources for Health to Achieve Resilient Health Systems” offers, through its strategic lines, a set of guidelines to support Member States in strengthening leadership and governance in human resources for health, updating regulatory frameworks, developing interprofessional teams, building competencies and ensuring decent work through adequate working conditions^(2)^.

In addition, the year 2025 presents a singular window of opportunity for actions aimed at strengthening the nursing workforce, with the publication of the State of the World’s Nursing report (SoWN) 2025, with current and relevant data on the nursing profile^(3)^, and the extension, until 2030, of the Global Strategic Directions for Nursing and Midwifery (SDNM), approved by the 78th World Health Assembly^(4)^. These documents provide evidence and recommendations for strategic actions aimed at the nursing workforce at the national level.

The global and regional agendas on the health workforce converge, among other issues, on the need to review and update the regulatory frameworks for the nursing profession, with a view to overcoming restrictive, often outdated policies and regulations that limit the scope of practice and the responsiveness of health systems, especially in keeping with the principles of PHC.

Several countries have reviewed and expanded the nursing scope of practice as a strategy to increase access to and coverage of healthcare, especially for populations in situations of vulnerability, in remote, rural and underserved areas. The adoption of expanded models of nursing practice in PHC reveals significant advances in the sharing of tasks between advanced practice nurses (APN) and other professionals, the expansion of timely access to health services and the reduction of pressure on health services, with the delivery of quality and safe care, provided by better qualified professionals^(5)^.

As a result, expanding nurses’ scope of practice is associated with greater PHC resolution and more efficient use of the system’s resources, with a positive impact on health promotion and disease prevention activities, continuity of care, a reduction in avoidable hospital admissions and greater user satisfaction^(5)^.

In the Region of the Americas, PAHO has been promoting the technical and political dialogue on expanding the scope of nursing practice for more than a decade, with a focus on implementing APN models in PHC. Countries such as Canada and the United States of America have had consolidated models since the 1960s, and significant progress has been made in several Caribbean countries, Brazil, Chile and Mexico, among others.

The experience of Latin American countries in developing and implementing the APN role highlights the importance of establishing intersectoral working groups, with broad participation of different stakeholders in the political dialogue for the effective and timely implementation of this role. However, progress is needed in terms of defining the APN competency profile, strengthening workforce governance, addressing the deficient regulatory framework and regulatory barriers, and debating the integration and adoption of this professional into the health labor market.

It’s not just a question of proposing and implementing the APN role, it also involves clearly defining the new professional roles and functions, regulating autonomy, establishing clinical protocols for their practice, debating the inclusion of these professionals in the health system, their remuneration policy and the acceptance of these professionals by health services, providers and the population.

More than just recognizing the central role of nursing in PHC, the challenges faced by the countries of the Region of the Americas require strategic and transformative actions. Updating regulatory frameworks and implementing innovative models of professional practice are concrete ways to strengthen PHC, expand equitable access and achieve better health outcomes.

Some strategies that would define the way forward in the integration and adoption of APN as a means to expand the scope of nursing practice include the following:

• Addressing professional, institutional, financial, cultural, structural and political barriers and advocating for policy changes that would address these obstacles;

• Investing in updating and strengthening education and training in terms of pursuing postgraduate degrees and qualifications not just of APN but also that of the nursing educators, including mentorship and continuous professional education;

• Developing leadership capacities to enhance their ability to lead teams, advocate for change, and participate in policy-making discussions to improve quality of care;

• Fostering collaboration and integration into interprofessional teams, working collaboratively with other health professionals to improve patient care and outcomes and with communities, expanding their reach beyond the traditional healthcare settings;

• Ensuring sustainability by building strong professional networks and proactive policies that promote research and innovation, while building public confidence in their capacities and recognition of their contributions to health systems.

Implementing these actions is also an investment in the future of the nursing profession. They will enable the preparation of future generations of nurses to support the transformation of health systems in the Region of the Americas, based on technical-scientific knowledge, social commitment and collaborative practice among interprofessional health teams.
