# Supplementary material for: Scope of Nursing Practice in Primary Health Care: defining a way forward
Source: Rev Lat Am Enfermagem. 2025 Nov 17;33:e4840. doi: 10.1590/1518-8345.0000.4840 (PMC12628368; doi:10.1590/1518-8345.0000.4840)
Supplement: Translation

# Translation: Escopo da Prática de Enfermagem na Atenção Primária à Saúde: definindo o caminho a seguir*

**DOI:** 10.1590/1518-8345.0000.4841

**Authors:** Bruna Moreno-Dias, Edwin Vicente C. Bolastig, Eduardo Benjamín Puertas-Donoso

**Affiliations:** 1 Pan American Health Organization, Department of Health Systems and Services, Human Resources for Health Unit, Washington, D.C., Estados Unidos da América.; 2 Pan American Health Organization, Subregional Program Coordination, Caribbean, Bridgetown, Barbados.

A Atenção Primária à Saúde (APS) é reconhecida como a base estrutural dos sistemas de saúde, uma estratégia abrangente para a organização e funcionamento do sistema de saúde, com o principal objetivo de alcançar a saúde universal. Os sistemas de saúde baseados na APS devem oferecer uma ampla gama de serviços integrais, acessíveis, equitativos, de alta qualidade e abrangentes, capazes de atender às necessidades de saúde de todas as pessoas ao longo de suas vidas, prestados por uma força de trabalho guiada pela prática colaborativa em equipes interprofissionais^(1-2)^.

Nesse contexto, a força de trabalho em saúde é um componente fundamental para a efetividade, equidade e resiliência dos sistemas de saúde. Destaca-se, também, o papel essencial desempenhado pelos profissionais de enfermagem, devido à sua presença ubíqua nos territórios, às suas relações com os usuários dos serviços e à amplitude de seu trabalho clínico^(2-3)^.

A Região das Américas conta com aproximadamente 7,4 milhões de profissionais de enfermagem; entretanto, enfrenta desafios persistentes relacionados à distribuição equitativa desses profissionais, à formação e qualificação dessa força de trabalho, à atualização dos marcos regulatórios e ao fortalecimento do escopo de prática em contextos complexos e desafiadores^(3)^.

Em resposta a esse cenário, a “Política sobre a força de trabalho em saúde para 2030: fortalecendo os recursos humanos em saúde para alcançar sistemas de saúde resilientes” da Organização Pan-Americana da Saúde (OPAS) oferece, por meio de suas linhas estratégicas, um conjunto de diretrizes para apoiar os Estados Membros no fortalecimento da liderança e governança dos recursos humanos em saúde, na atualização dos marcos regulatórios, no desenvolvimento de equipes interprofissionais, na construção de competências e na garantia de trabalho decente por meio de condições adequadas de trabalho^(2)^.

Além disso, o ano de 2025 apresenta uma janela singular de oportunidade para ações voltadas ao fortalecimento da força de trabalho em enfermagem, com a publicação do relatório *State of the World’s Nursing* (SoWN) 2025, contendo dados atuais e relevantes sobre o perfil da enfermagem^(3)^, e a prorrogação, até 2030, das Diretrizes Estratégicas Globais para Enfermagem e Obstetrícia (SDNM), aprovadas pela 78ª Assembleia Mundial da Saúde^(4)^. Esses documentos fornecem evidências e recomendações para ações estratégicas direcionadas à força de trabalho em enfermagem no âmbito nacional.

As agendas global e regional sobre a força de trabalho em saúde convergem, entre outros aspectos, sobre a necessidade de revisar e atualizar os marcos regulatórios da profissão de enfermagem, com vistas a superar políticas e regulamentos restritivos, muitas vezes desatualizados, que limitam o escopo de prática e a capacidade de resposta dos sistemas de saúde, especialmente em consonância com os princípios da APS.

Diversos países revisaram e ampliaram o escopo da prática de enfermagem como estratégia para aumentar o acesso e a cobertura dos serviços de saúde, especialmente para populações em situação de vulnerabilidade, em áreas remotas, rurais e desassistidas. A adoção de modelos ampliados de prática de enfermagem na APS revela avanços significativos no compartilhamento de tarefas entre enfermeiros de prática avançada (EPA) e outros profissionais, na ampliação do acesso oportuno aos serviços de saúde e na redução da pressão sobre os serviços, com a oferta de cuidados de qualidade e segurança, prestados por profissionais mais qualificados^(5)^.

Como resultado, a ampliação do escopo da prática de enfermagem associa-se a maior resolutividade na APS e ao uso mais eficiente dos recursos do sistema, com impacto positivo nas atividades de promoção da saúde e prevenção de doenças, continuidade do cuidado, redução de hospitalizações evitáveis e maior satisfação dos usuários^(5)^.

Na Região das Américas, a OPAS tem promovido, há mais de uma década, o diálogo técnico e político sobre a ampliação do escopo da prática de enfermagem, com foco na implementação de modelos de EPA na APS. Países como Canadá e Estados Unidos da América possuem modelos consolidados desde a década de 1960, e avanços significativos foram alcançados em diversos países do Caribe, Brasil, Chile e México, entre outros.

A experiência dos países latino-americanos no desenvolvimento e implementação do papel do EPA destaca a importância de estabelecer grupos de trabalho intersetoriais, com ampla participação de diferentes atores no diálogo político, para a implementação efetiva e oportuna desse papel. No entanto, ainda são necessários avanços quanto à definição do perfil de competências do EPA, ao fortalecimento da governança da força de trabalho, à superação de marcos regulatórios deficientes e barreiras normativas, e ao debate sobre a integração e adoção desse profissional no mercado de trabalho em saúde.

Não se trata apenas de propor e implementar o papel do EPA, mas também de definir claramente os novos papéis e funções profissionais, regular a autonomia, estabelecer protocolos clínicos para sua prática, debater sua inclusão no sistema de saúde, a política de remuneração e a aceitação desses profissionais pelos serviços, provedores e pela população.

Mais do que reconhecer o papel central da enfermagem na APS, os desafios enfrentados pelos países da Região das Américas exigem ações estratégicas e transformadoras. A atualização dos marcos regulatórios e a implementação de modelos inovadores de prática profissional são caminhos concretos para fortalecer a APS, ampliar o acesso equitativo e alcançar melhores resultados em saúde.

Algumas estratégias que definem o caminho a seguir na integração e adoção do EPA como meio para expandir o escopo da prática de enfermagem incluem:

• Enfrentar barreiras profissionais, institucionais, financeiras, culturais, estruturais e políticas, e advogar por mudanças de políticas que as superem;

• Investir na atualização e fortalecimento da educação e formação, promovendo cursos de pós-graduação e qualificações não apenas para os EPA, mas também para os educadores em enfermagem, incluindo mentoria e educação profissional contínua;

• Desenvolver capacidades de liderança para ampliar sua habilidade de conduzir equipes, advogar por mudanças e participar de discussões de formulação de políticas para melhorar a qualidade do cuidado;

• Fomentar a colaboração e integração em equipes interprofissionais, trabalhando em conjunto com outros profissionais da saúde e com as comunidades, ampliando seu alcance para além dos espaços tradicionais de cuidado;

• Garantir a sustentabilidade por meio da construção de redes profissionais sólidas e políticas proativas que promovam pesquisa e inovação, fortalecendo a confiança pública em suas capacidades e o reconhecimento de suas contribuições aos sistemas de saúde.

A implementação dessas ações também constitui um investimento no futuro da profissão de enfermagem. Elas permitirão preparar as futuras gerações de enfermeiros para apoiar a transformação dos sistemas de saúde na Região das Américas, com base no conhecimento técnico-científico, no compromisso social e na prática colaborativa em equipes interprofissionais de saúde.

# Translation: Ámbito de la Práctica de Enfermería en la Atención Primaria de Salud: definiendo el camino a seguir*

**DOI:** 10.1590/1518-8345.0000.4839

**Authors:** Bruna Moreno-Dias, Edwin Vicente C. Bolastig, Eduardo Benjamín Puertas-Donoso

**Affiliations:** 1 Pan American Health Organization, Department of Health Systems and Services, Human Resources for Health Unit, Washington, D.C., Estados Unidos da América.; 2 Pan American Health Organization, Subregional Program Coordination, Caribbean, Bridgetown, Barbados.

La Atención Primaria de Salud (APS) es reconocida como la base estructural de los sistemas de salud, una estrategia integral para la organización y funcionamiento del sistema de salud, cuyo objetivo principal es alcanzar la salud universal. Los sistemas de salud basados en la APS deben ofrecer una amplia gama de servicios integrales, accesibles, equitativos, de alta calidad y completos para satisfacer las necesidades de salud de todas las personas a lo largo de su vida, prestados por una fuerza laboral guiada por la práctica colaborativa en equipos interprofesionales^(1-2)^.

En este contexto, la fuerza laboral en salud constituye un componente fundamental para la eficacia, la equidad y la resiliencia de los sistemas de salud. También se destaca el papel esencial que desempeña el personal de enfermería, debido a su presencia ubicua en los territorios, sus relaciones con los usuarios de los servicios y la amplitud de su labor clínica^(2-3)^.

La Región de las Américas cuenta con aproximadamente 7,4 millones de profesionales de enfermería; sin embargo, enfrenta desafíos persistentes relacionados con la distribución equitativa de estos profesionales, la formación y calificación de esta fuerza laboral, la actualización de los marcos regulatorios y el fortalecimiento del ámbito de la práctica en contextos complejos y desafiantes^(3)^.

En respuesta a este escenario, la “Política sobre el personal de salud 2030: fortalecimiento de los recursos humanos para la salud a fin de lograr sistemas de salud resilientes” de la Organización Panamericana de la Salud (OPS) ofrece, a través de sus líneas estratégicas, un conjunto de directrices que apoyan a los Estados Miembros en el fortalecimiento del liderazgo y la gobernanza de los recursos humanos en salud, la actualización de los marcos regulatorios, el desarrollo de equipos interprofesionales, la construcción de competencias y la garantía de trabajo decente mediante condiciones laborales adecuadas^(2)^.

Además, el año 2025 representa una oportunidad única para impulsar acciones orientadas al fortalecimiento del personal de enfermería, con la publicación del informe *State of the World’s Nursing* (SoWN) 2025, con datos actuales y relevantes sobre el perfil de la enfermería^(3)^, y la extensión, hasta 2030, de las Orientaciones estratégicas mundiales sobre enfermería y partería (SDNM), aprobadas por la 78ª Asamblea Mundial de la Salud^(4)^. Estos documentos proporcionan evidencias y recomendaciones para acciones estratégicas dirigidas al personal de enfermería a nivel nacional.

Las agendas global y regional sobre la fuerza laboral en salud coinciden, entre otros aspectos, en la necesidad de revisar y actualizar los marcos regulatorios de la profesión de enfermería, con el fin de superar políticas y regulaciones restrictivas, muchas veces obsoletas, que limitan el ámbito de la práctica y la capacidad de respuesta de los sistemas de salud, especialmente en consonancia con los principios de la APS.

Varios países han revisado y ampliado el ámbito de la práctica de enfermería como estrategia para aumentar el acceso y la cobertura de los servicios de salud, especialmente para poblaciones en situación de vulnerabilidad, en áreas remotas, rurales y desatendidas. La adopción de modelos ampliados de práctica de enfermería en la APS muestra avances significativos en la redistribución de tareas entre las enfermeras de práctica avanzada (EPA) y otros profesionales, la ampliación del acceso oportuno a los servicios de salud y la reducción de la presión sobre dichos servicios, ofreciendo atención segura y de calidad prestada por profesionales mejor calificados^(5)^.

Como resultado, la ampliación del ámbito de la práctica de enfermería se asocia con una mayor capacidad resolutiva en la APS y un uso más eficiente de los recursos del sistema, con impacto positivo en las actividades de promoción de la salud y prevención de enfermedades, la continuidad de la atención, la reducción de hospitalizaciones evitables y una mayor satisfacción de los usuarios^(5)^.

En la Región de las Américas, la OPS ha promovido durante más de una década el diálogo técnico y político sobre la ampliación del ámbito de la práctica de enfermería, con enfoque en la implementación de modelos de EPA en la APS. Países como Canadá y los Estados Unidos de América cuentan con modelos consolidados desde la década de 1960, y se han logrado avances significativos en varios países del Caribe, Brasil, Chile y México, entre otros.

La experiencia de los países latinoamericanos en el desarrollo e implementación del rol de la EPA resalta la importancia de establecer grupos de trabajo intersectoriales, con amplia participación de diversos actores en el diálogo político, para la implementación efectiva y oportuna de este rol. No obstante, se requieren avances en la definición del perfil de competencias de la EPA, el fortalecimiento de la gobernanza de la fuerza laboral, la superación de los marcos regulatorios deficientes y las barreras normativas, así como el debate sobre la integración y adopción de este profesional en el mercado laboral de la salud.

No se trata solo de proponer e implementar el rol de la EPA, sino también de definir claramente los nuevos roles y funciones profesionales, regular su autonomía, establecer protocolos clínicos para su práctica, debatir su inclusión en el sistema de salud, su política de remuneración y la aceptación de estos profesionales por parte de los servicios, los proveedores y la población.

Más allá de reconocer el papel central de la enfermería en la APS, los desafíos que enfrentan los países de la Región de las Américas requieren acciones estratégicas y transformadoras. La actualización de los marcos regulatorios y la implementación de modelos innovadores de práctica profesional constituyen caminos concretos para fortalecer la APS, ampliar el acceso equitativo y alcanzar mejores resultados en salud.

Algunas estrategias que definen el camino a seguir en la integración y adopción de la EPA como medio para ampliar el ámbito de la práctica de enfermería incluyen:

• Abordar las barreras profesionales, institucionales, financieras, culturales, estructurales y políticas, y abogar por cambios normativos que las eliminen;

• Invertir en la actualización y fortalecimiento de la educación y la formación, promoviendo estudios de posgrado y calificaciones no solo para las EPA, sino también para los educadores en enfermería, incluyendo mentoría y educación profesional continua;

• Desarrollar capacidades de liderazgo para fortalecer su habilidad de dirigir equipos, abogar por el cambio y participar en discusiones sobre políticas públicas para mejorar la calidad del cuidado;

• Fomentar la colaboración e integración en equipos interprofesionales, trabajando conjuntamente con otros profesionales de la salud y con las comunidades, ampliando su ámbito más allá de los entornos tradicionales de atención;

• Garantizar la sostenibilidad mediante la construcción de redes profesionales sólidas y políticas proactivas que promuevan la investigación y la innovación, fortaleciendo la confianza pública en sus capacidades y el reconocimiento de sus contribuciones a los sistemas de salud.

La implementación de estas acciones también representa una inversión en el futuro de la profesión de enfermería. Permitirán preparar a las futuras generaciones de enfermeras para apoyar la transformación de los sistemas de salud en la Región de las Américas, basándose en el conocimiento técnico-científico, el compromiso social y la práctica colaborativa entre equipos interprofesionales de salud.
